# Unsalted tomato juice intake improves blood pressure and serum low‐density lipoprotein cholesterol level in local Japanese residents at risk of cardiovascular disease

**DOI:** 10.1002/fsn3.1066

**Published:** 2019-05-15

**Authors:** Tamami Odai, Masakazu Terauchi, Daisaku Okamoto, Asuka Hirose, Naoyuki Miyasaka

**Affiliations:** ^1^ Department of Obstetrics and Gynecology Tokyo Medical and Dental University Tokyo Japan; ^2^ Department of Women's Health Tokyo Medical and Dental University Tokyo Japan; ^3^ Plant Breeding Institute Co., Ltd Kuriyama, Yubari Japan

**Keywords:** cardiovascular diseases, hypertension, prehypertension, dyslipidemia, hypercholesterolemia

## Abstract

The aim of this study was to investigate the effects of unsalted tomato juice intake on cardiovascular risk markers in local Japanese residents. Four hundred and eighty‐one local residents in Kuriyama, Japan, were enrolled in this study. Throughout the year of the study, they were provided with as much unsalted tomato juice as they wanted. Participants were screened for cardiovascular risk markers, such as blood pressure (BP), serum lipid profile, and glucose tolerance, before and after the study period. Of the study participants, 260 participated in a detailed study of their lifestyle factors. The average ages of the 184 male and 297 female participants were 56.3 ± 13.3 (mean ± *SD*) and 58.4 ± 11.7 years, respectively. BP in 94 participants with untreated prehypertension or hypertension was significantly lowered (systolic BP, 141.2 ± 12.1–137.0 ± 16.3 mmHg, *p* = 0.003; diastolic BP, 83.3 ± 10.1–80.9 ± 11.1 mmHg, *p* = 0.012, paired *t* test). Further, the serum low‐density lipoprotein cholesterol (LDL‐C) level in 125 participants with untreated dyslipidemia significantly decreased (155.0 ± 23.2–149.9 ± 25.0 mg/dl, *p* = 0.005, paired *t* test). These beneficial effects were not different between sexes and among the different age groups. No significant difference in lifestyle was found before and after the study. Unsalted tomato juice intake improved systolic and diastolic BP and serum LDL‐C level in local Japanese residents at risk of cardiovascular conditions.

## 1 INTRODUCTION

Cardiovascular diseases (CVDs) are the biggest causes of mortality worldwide, and according to the World Health Organization (WHO), they were responsible for 15.2 million global deaths (26.7%) in 2016 (retrieved from http://www.who.int/mediacentre/factsheets/fs310/en/). Even when CVDs are not fatal, they often result in permanent damage to critical organs, which in turn causes activity restriction, nursing care, and reduced life expectancy. The main pathophysiological cause of CVDs is atherosclerosis, which is a chronic inflammatory reaction that begins as a response to injury of the arterial intima (Ross, 1999). This type of endothelial injury is induced by several factors, such as endotoxins, viruses, homocysteine, and cigarette smoke (Widlansky, Gokce, Keaney, & Vita, 2003). Chronic endothelial injury in hypertension, dyslipidemia, and diabetes are also important contributors of atherosclerosis progression (National Heart, Lung, & Blood Institute, 2013 retrieved from https://www.nhlbi.nih.gov/health-topics/assessing-cardiovascular-risk, Beckman, Creager, & Libby, 2002; Libby, Aikawa, & Schönbeck, 2000; Sander, Kukla, Klingelhöfer, Winbeck, & Conrad, 2000). Therefore, it is crucial to regulate blood pressure (BP), and lipid and glucose metabolism, to prevent the development of CVDs.

Tomato contains a variety of bioactive compounds, such as carotenoid, vitamin A, calcium, and gamma‐aminobutyric acid, which may play a role in maintaining physical and psychological health, including the prevention of CVD (Hak et al., 2004; Yanai et al., 2017; Zorumski, Paul, Izumi, Covey, & Mennerick, 2013). For example, the intake of lycopene, a carotenoid rich in tomatoes and known to have strong antioxidant activity (Oshima, Ojima, Sakamoto, Ishiguro, & Terao, 1996), has been reported to be inversely associated with the risk of CVDs (Agarwal & Rao, 2000; Hak et al., 2004; Rissanen et al., 2001); the mechanism underlying this effect may be an improvement in the serum lipid profile (Ried & Falker, 2011; Sesso, Wang, Ridker, & Buring, 2012; Yanai et al., 2017). There are also reports about the beneficial effects of lycopene on BP (Engelhard, Gazer, & Paran, 2006; Paran, Novack, Engelhard, & Hazan‐Halevy, 2009; Ried & Falker, 2011; Yanai et al., 2017). Esculeoside A, a saponin found in tomatoes, has also been reported to suppress the activity of acyl‐CoA: cholesterol acyltransferase (ACAT), leading to an improvement in dyslipidemia (Nohara, 2010). Furthermore, 13‐oxo‐9, 11‐octadecadienoic acid (13‐oxo‐ODA), a conjugated linoleic acid newly identified in tomato juice, was shown to have antidyslipidemic effects (Kim et al., 2012). Recently, we reported that unsalted tomato juice intake for 8 weeks improved hypertriglyceridemia in middle‐aged Japanese women (Hirose et al., 2015), which prompted us to investigate the effects of tomato juice on cardiovascular risk markers, such as BP, and lipid and glucose metabolism, in local Japanese male and female residents over a wider age range in this study.

## 2 MATERIALS AND METHODS

### 2.1 Study population

**Figure 1:**
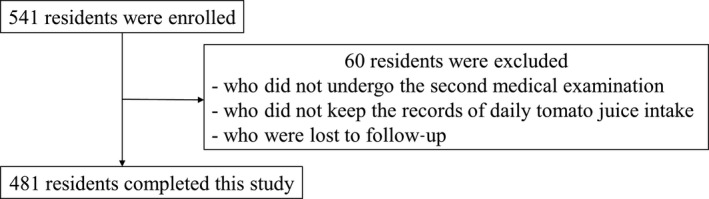
Study flowchart

The participants of the present study were recruited from among the local residents in Kuriyama, Hokkaido, Japan. We sent flyers of this study to the local residents aged 20–74 years and held several briefing sessions to the candidates to recruit as many participants as possible. The participants received as much unsalted tomato juice (Nippon Del Monte) as they wanted throughout the year of the study. The nutritional composition of the tomato juice used in the present study, which was the same product as that was used in the previous study, is shown in Table 1 (Hirose et al., 2015). Participants kept records of daily tomato juice consumption, as well as any medical treatment and changes therein. We collected the participants’ diaries every 3 months and calculated overall tomato juice consumption and total days of tomato juice intake throughout the study period based on the diaries, and then calculated the frequency of tomato juice intake and the average tomato juice consumption per day across the entire cohort. Five hundred and forty‐one residents, who accounted for 4.3% of the population of Kuriyama, were enrolled, and 481 (88.9%) completed this study (Figure 1).

**Table 1:** The nutritional composition of the unsalted tomato juice used in the present study

Nutrient	Value per 200 ml (1 bottle)
Energy (kilocalories)	41
Protein (g)	2.2
Fat (g)	0
Sugars (g)	7.2
Dietary fiber (g)	1.8
Sodium (mg)	16
Calcium (mg)	23
Potassium (mg)	630
Vitamin A (μg)	46
GABA (mg)	99
Lycopene (mg)	22
13‐oxo‐ODA (μg)	39.2
Esculeoside A	Unknown

The study protocol was reviewed and approved by the Tokyo Medical and Dental University Review Board, and written informed consent was obtained from all participants. The study was conducted in accordance with the Declaration of Helsinki.

### 2.2 Measurement

The participants underwent an annual medical checkup, which was conducted according to the public policy of the Japanese government to prevent lifestyle‐related diseases, before and after the study period. Physical examination was conducted by local government or medical institutions designated by the health insurance system according to the protocol provided by the Japanese Ministry of Health, Labor and Welfare. The medical examination included height, weight, abdominal circumference, systolic BP (SBP) and diastolic BP (DBP), and serum levels of triglyceride (TG), high‐density lipoprotein cholesterol (HDL‐C), low‐density lipoprotein cholesterol (LDL‐C), fasting plasma glucose (FPG), and hemoglobin A1c (HbA1c). Hypertension and impaired lipid and glucose metabolism were defined according to the diagnostic criteria in Japan. BP was categorized as normal (SBP <130 mmHg and DBP <85 mmHg), prehypertension (SBP, 130–139 mmHg and/or DBP, 85–89 mmHg), and hypertension (SBP ≧140 mmHg and/or DBP ≧90 mmHg). Dyslipidemia was categorized as hypertriglyceridemia (TG ≧150 mg/dl), hypo‐HDL cholesterolemia (HDL <40 mg/dl), and hyper‐LDL cholesterolemia (LDL‐C ≧140 mg/dl). Participants who had FPG level ≧126 mg/dl and HbA1c ≧6.5% were diagnosed with type 2 diabetes, and those with an FPG level 110–125 mg/dl and/or HbA1c 6.0%–6.4% were considered to have impaired glucose tolerance.

### 2.3 Lifestyle factors

Of the 481 study participants, only 260 (54.1%) who underwent the medical checkup performed by the local government of Kuriyama participated in a detailed study of their lifestyle factors. This involved completing a questionnaire defined by the Japanese Ministry of Health, Labor and Welfare, before and after the intervention (Table 2). The questionnaire was composed of 22 questions, including past medical history, concurrent therapeutic agents, and lifestyle factors, such as diet, exercise, and smoking. Ten of these questions, namely those about exercise, dietary habits, change in body weight, sleeping, and smoking, were assessed with regard to possible lifestyle alterations.

**Table 2:** Questionnaire

No	Questions
1–3	Are you currently taking the following medications?
a	A drug to lower blood pressure
b	Insulin injections or a drug to lower blood glucose
c	A drug to lower cholesterol
4	Have you ever had stroke or have you ever received treatment for stroke?
5	Have you ever had heart disease or have you ever received treatment for heart disease?
6	Have you ever had chronic renal failure or have you ever received treatment for chronic renal failure (dialysis)?
7	Have you ever had anemia?
8	Are you a current regular smoker?
9	Have you gained 10 kg or more since you were 20 years old?
10	Have you been exercising at least 2 days per week, at 30 min each at an intensity that causes a slight sweat, for at least 1 year?
11	Do you walk for at least 1 hr every day or have equivalent physical activities in your daily life?
12	Do you walk faster than people of your age and sex?
13	Have you had a weight gain or loss of 3 kg or more over the last year?
14	How fast do you eat compared to others?
15	Do you have an evening meal within 2 hr before bedtime 3 days or more per week?
16	Do you eat after the evening meal 3 days or more per week?
17	Do you skip breakfast 3 days or more per week?
18	How often do you drink alcohol?
19	How much do you drink a day, in terms of glasses of refined sake?
20	Do you feel refreshed after a night's sleep?
21	Are you going to start or have you started lifestyle modifications?
22	Are you willing to get health counseling about lifestyle modifications if the opportunity arises?

Note

Defined by the Japanese Ministry of Health, Labor and Welfare.

### 2.4 Statistical analyses

Statistical analyses were performed with GraphPad Prism version 5.0 (GraphPad Software). Paired *t* test was used to compare the results of physical and blood examination before and after the intervention, and differences between men and women were examined by unpaired *t* test. Changes in CVD risk markers among different age groups were compared with one‐way analysis of variance (ANOVA), and changes in lifestyle were evaluated by chi‐square test. *p* values <0.05 were considered statistically significant.

## 3 RESULTS

### 3.1 Participants and tomato juice consumption

**Figure 2:**
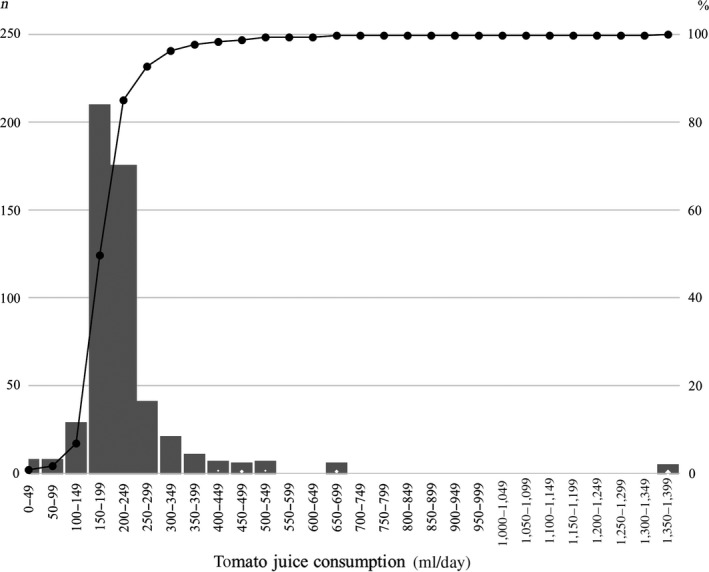
Average daily tomato juice consumption and cumulative frequency: The number of participants for each segment of daily tomato juice intake (*x*‐axis) is represented in bars on the left‐hand axis. The line and right‐hand axis show the cumulative percentage

Four hundred and eighty‐one residents were enrolled, including 184 men and 297 women, aged 21–74 years. The average age (years) of men and women was 56.3 ± 13.3 (mean ± *SD*) and 58.4 ± 11.7, respectively. Among all participants, the frequency of daily unsalted tomato juice intake throughout the study period was 92.0 ± 12.2%, and the average daily tomato juice consumption was 215 ± 84 ml. Most of the study participants consumed about 1 bottle (200 ml) of unsalted tomato juice every day (Figure 2). Nine participants who consumed <100 ml/day tomato juice were excluded from further analysis. Table 3 presents the overall results of physical and blood examination in 481 participants before and after the intervention. No significant differences were found in any factor before and after the study period.

**Table 3:** Results of physical and blood examination in 481 participants before and after the study period

	Before the intervention		After the intervention		*n*
	Mean	*SD*	Mean	*SD*	
Body mass index, kg/m^2^	23.3	3.5	23.4	3.5	481
Abdominal circumference, cm	81.5	9.5	81.3	9.6	469
Systolic blood pressure, mmHg	125.1	17.3	124.9	16.8	480
Diastolic blood pressure, mmHg	74.7	11.0	74.4	10.8	480
Triglyceride, mg/dl	102.3	56.8	106.1	64.9	481
High‐density lipoprotein cholesterol, mg/dl	65.3	16.8	65.4	16.4	481
Low‐density lipoprotein cholesterol, mg/dl	123.6	29.6	123.2	29.3	474
Fasting plasma glucose, mg/dl	95.8	16.6	95.3	13.8	430
Hemoglobin A1c, %	5.6	0.5	5.7	0.5	407

Note

Data are presented as the mean and *SD*. There were no significant differences in any factor before and after the intervention.

### 3.2 Participants at risks of CVDs

**Figure 3:**
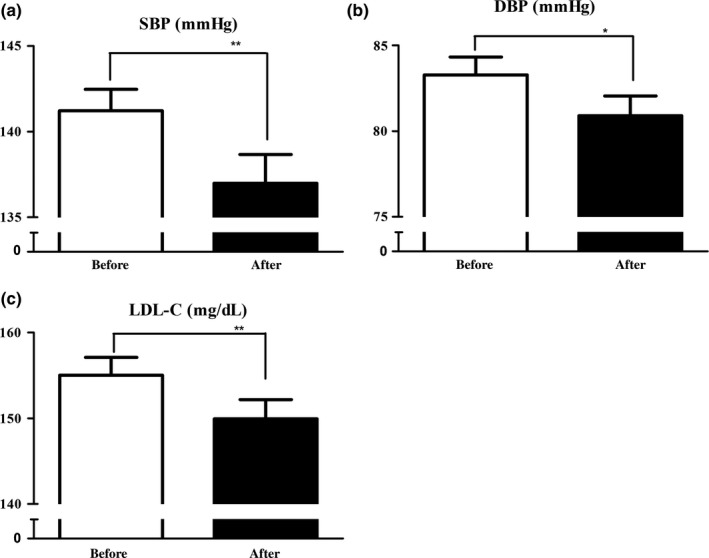
Systolic and diastolic blood pressure, and the serum LDL‐C level before and after the intervention: (a) systolic and (b) diastolic blood pressure in 94 participants with untreated prehypertension or hypertension; (c) the serum levels of LDL‐C in 125 participants with untreated dyslipidemia. Data are presented as the standard error of the mean. *p < 0.05, ** < 0.01, versus before the intervention, paired *t* test

Next, we compared BP before and after the study period in 94 participants with untreated prehypertension or hypertension. The mean SBP and DBP were significantly lowered after a year of tomato juice intake (SBP, 141.2 ± 12.1–137.0 ± 16.3 mmHg, *p* = 0.003; DBP, 83.3 ± 10.1–80.9 ± 11.1 mmHg, *p* = 0.012, paired *t* test) (Figure 3a,b). In 127 participants with untreated dyslipidemia, the mean serum TG and HDL‐C level did not change significantly after a year of tomato juice consumption (TG, 130.3 ± 69.6–136.8 ± 84.2 mg/dl, *p* = 0.255; HDL‐C, 63.0 ± 17.3–61.6 ± 16.6 mg/dl, *p* = 0.051), whereas the mean serum LDL‐C level was significantly decreased (155.0 ± 23.2–149.9 ± 25.0 mg/dl, *p* = 0.005, paired *t* test) (Figure 3c). We also evaluated glucose metabolism in 62 participants with untreated impaired glucose tolerance. No statistically significant change in FPG level and HbA1c was observed after the study period (FPG, 107.8 ± 11.6–107.2 ± 11.7 mg/dl, *p* = 0.686; HbA1c, 6.1 ± 0.4–6.1 ± 0.4%, *p* = 0.385).

### 3.3 Difference between sexes

Next, we compared the effects of unsalted tomato juice intake on SBP, DBP, and serum LDL‐C level between men and women. The 94 participants with untreated prehypertension or hypertension included 43 men and 51 women aged 23–74 years, and the 127 participants with untreated dyslipidemia included 52 men and 73 women, ranging from 25 to 74 years. There were no significant differences in the mean changes in SBP, DBP, and serum LDL‐C level between the sexes (SBP, −4.1 ± 15.0 and −4.3 ± 12.4 mmHg, *p* = 0.939; DBP, −1.52 ± 9.4 and −3.2 ± 8.6 mmHg, *p* = 0.362; LDL‐C, −4.8 ± 22.7 and −5.3 ± 18.0 mg/dl, *p* = 0.480, men and women, unpaired *t* test).

### 3.4 Difference among age groups

We also compared the changes in SBP, DBP, and the serum level of LDL‐C before and after the intervention among different age groups. The 94 participants with untreated prehypertension or hypertension were divided into three age groups, namely young (23–54 years, *n* = 28), middle‐aged (55–64 years, *n* = 33), and old (65–74 years, *n* = 33). No statistically significant differences in the mean changes in SBP and DBP were observed among the groups (SBP: young, −0.2 ± 13.2; middle‐aged, −5.4 ± 15.1; old, −6.6 ± 11.7 mmHg; *p* = 0.155, one‐way ANOVA, and DBP: young, −0.8 ± 9.4; middle‐aged, −1.7 ± 9.6; old, −4.3 ± 7.8 mmHg; *p* = 0.281, respectively). Dividing the 127 participants with untreated dyslipidemia into young (*n* = 39), middle‐aged (*n* = 43), and old (*n* = 43) in the same way, the mean changes in serum LDL‐C level were not different significantly among the groups (young, −5.0 ± 21.4; middle‐aged, −3.9 ± 18.6; old, −6.3 ± 20.5 mg/dl; *p* = 0.854, one‐way ANOVA).

### 3.5 Change in lifestyle factors

Finally, we investigated whether or not the improvements in cardiovascular markers in participants at risk could be attributable to changes in lifestyle during the study period. Among the 260 residents who participated in the detailed lifestyle study, 40 had untreated prehypertension or hypertension, and 69 had untreated dyslipidemia. The lifestyle factors did not differ significantly between the groups either before or after the study period (chi‐square test) (Table 4).

**Table 4:** Change in lifestyle factors

	Untreated prehypertension or hypertension			Untreated dyslipidemia		
	Before (*n*)	After (*n*)	*p* value	Before (*n*)	After (*n*)	*p* value
Exercise						
Yes	8	32	0.439	18	13	0.415
No	12	28		51	56	
Physical activity						
Yes	17	23	1.000	25	32	0.300
No	16	24		44	37	
Speed of walking						
Fast	22	15	0.178	33	32	1.000
Normal	18	24		35	36	
Speed of eating						
Fast	14	14	1.000	20	18	0.706
Normal/Slow	26	26		48	51	
Eating before bedtime						
Yes	2	4	0.675	9	7	0.791
No	38	36		60	62	
Snack between meals						
Yes	4	5	1.000	8	4	0.366
No	36	35		61	65	
Skip breakfast						
Yes	1	2	1.000	4	7	0.532
No	39	38		64	62	
Change in body weight						
Yes	6	6	1.000	14	15	1.000
No	33	34		55	54	
Sleeping						
Good	33	36	0.518	55	54	0.833
Bad	7	4		13	15	
Smoking						
Yes	7	4	1.000	13	15	0.833
No	33	36		55	54	

Note

Data show the change of lifestyle factors in 40 participants with untreated prehypertension or hypertension and in 69 with untreated dyslipidemia before and after the study period. No significant differences in lifestyle factors were found between the groups (chi‐square test).

## 4 DISCUSSION

The present study showed that unsalted tomato juice intake may have contribution to lower SBP and DBP in local Japanese residents who had untreated prehypertension or hypertension, and improve serum LDL‐C level in those who had untreated dyslipidemia. These ameliorative effects were not different between sexes and different age groups and could not be attributable to the alteration in lifestyle. To the best of our knowledge, the current study is the first to investigate the effects of tomato or tomato product intake on CVD risk markers over the course of a year and over a wide age range.

Tomatoes contain a variety of bioactive components that make them and their products, including tomato juice, beneficial for health (Engelhard et al., 2006; Hsu et al., 2008; Oshima et al., 1996; Paran et al., 2009; Ried & Falker, 2011; Sesso et al., 2012). Above all, lycopene is well known for its strong antioxidant activity and the inhibition of LDL oxidation, which plays a key role in the initiation and development of atherosclerosis. Several epidemiological studies have suggested that lycopene could contribute to the prevention of atherosclerosis and CVDs (Hak et al., 2004; Klipstein‐Grobusch et al., 2000; Kohlmeier et al., 1997; Rissanen et al., 2003). For example, the serum lycopene concentration was inversely associated with calcified deposits in the abdominal aorta (Klipstein‐Grobusch et al., 2000) and the intima‐media thickness of the common carotid artery (Rissanen et al., 2003). Recently, novel molecular mechanisms underlying lycopene's ability to prevent atherosclerosis have been identified: These include the regulation of cholesterol metabolism by lycopene through the suppression of cholesterol synthesis and efflux in macrophages (Palozza, Parrone, Simone, & Catalano, 2010; Palozza, Simone, Catalano, Parrone, et al., 2011). The anti‐inflammatory effects of lycopene in the atherosclerosis process have also been shown (Palozza et al., 2010; Palozza, Simone, Catalano, Monego, et al., 2011). Studies suggested that lycopene could reduce the production of proinflammatory cytokines through activation of peroxisome proliferator‐activated receptor γ and inhibition of nuclear factor‐κB. Furthermore, the beneficial effects on BP (Engelhard et al., 2006; Palozza, Simone, Catalano, Monego, et al., 2011; Palozza, Simone, Catalano, Parrone, et al., 2011; Paran et al., 2009) and the ameliorative effects of endothelial function by the antioxidant activity of lycopene have been reported (Gajendragadkar et al., 2014; Kim, Paik, et al., 2011; Zhu, Wang, & Xu, 2011). Although the mechanism is yet to be elucidated, our study showed the beneficial effects of unsalted tomato juice intake on SBP, DBP, and LDL‐C; thus, our findings may support these reports of the beneficial properties of lycopene.

Esculeoside A, a tomato saponin, has also been suggested to have protective effects on dyslipidemia and atherosclerosis development by inhibiting ACAT (Fujiwara et al., 2007; Nohara, Ono, Ikeda, Fujiwara, & El‐Asar, 2010). Inhibition of ACAT decreases the absorption of diet‐derived cholesterol in the small intestine, macrophage foam cell formation, and cholesterol synthesis in the liver. In 2007, Fujiwara et al. demonstrated that esculeoside A suppressed the activity of ACAT, reduced the serum levels of TG, LDL‐C, and total cholesterol, and improved atherosclerotic lesions in apolipoprotein E‐deficient mice (Fujiwara et al., 2007). Esculeoside A could have played a role in improving serum LDL‐C level in our study participants although its amount in the tomato juice used in the current study was unknown.

Peroxisome proliferator‐activated receptors (PPARs), of which three subtypes have been identified (α, β, γ), regulate energy homeostasis, including lipid and glucose metabolism (Braissant, Foufelle, Scotto, Dauca, & Wahli, 1996; Varga, Czimmerer, & Nagy, 2011; Wahli, Braissant, & Desvergne, 1995). PPARα is strongly expressed in tissues with a high mitochondrial and peroxisomal β‐oxidation activity, such as the heart, liver, kidney, and intestine (Braissant et al., 1996), and activation of PPARα results in enhancement of fatty acid oxidation and improvement of dyslipidemia (Wahli et al., 1995). Oxo‐octadecadienoic acid (oxo‐ODA) found in tomato was recently reported to act as a PPARα agonist ( Kim, Hirai, et al., 2011; Kim et al., 2012; Takahashi et al., 2011). Although four structural isomers of oxo‐ODA were identified that activate PPARα equally (Braissant et al., 1996; Kim et al., 2012), 9‐oxo‐10(E),12(E)‐ODA and 13‐oxo‐ODA were the only ones detected in tomato juice (Takahashi et al., 2011). Kim et al. (2012) showed that 9‐oxo‐10(E),12(E)‐ODA decreased cellular accumulation of TG in mice hepatocytes, and 13‐oxo‐ODA reduced plasma and hepatic TG level and plasma glucose level in obese diabetic mice. PPARα activation also plays a role in antioxidant and anti‐inflammatory effects (Delerive, Gervois, Fruchart, & Staels, 2000; Deplanque et al., 2003; Ibarra‐Lara et al., 2010, 2012; Varga et al., 2011). It has been suggested that PPARα stimulation induces antioxidant activity, leading to improvement in the activity of endothelial factors that control blood pressure (Ibarra‐Lara et al., 2010) and cardiac function (Ibarra‐Lara et al., 2012), and neuroprotective effects against cerebral injury (Deplanque et al., 2003). Furthermore, the atherosclerosis protective effects of PPARα activation are also thought to occur through enhancement of anti‐inflammatory response (Cao, Wen, & Li, 2014). The regulation of energy metabolism, oxidative stress, and the inflammatory response by activation of PPARα is expected to lead to the prevention of atherosclerosis and CVDs. In our study, oxo‐ODAs in tomato juice could have contributed to the ameliorative effects on the serum LDL‐C level and blood pressure.

The present study has some limitations. Firstly, as most of the study participants consumed about one bottle of tomato juice (200 ml) every day, we could not evaluate whether the effects of tomato juice on CVD risk markers depended on the level of consumption. Secondly, the detailed information of the diet each participant followed during the study period is lacking. We also did not assess the intake of other juices and nutritional supplements. Finally, the study on lifestyle factors was conducted in only about half of the participants and the findings may not be applicable to the whole study population.

In conclusion, our study shows that unsalted tomato juice intake could have improved systolic and diastolic blood pressure in Japanese residents who had untreated prehypertension or hypertension, and also decreased the serum LDL‐C level in those who had untreated dyslipidemia. As tomato juice is an affordable and readily product, it could be practical as applied a nutritional intervention to prevent CVDs in people at risk.

## CONFLICT OF INTEREST

MT received an unrestricted research grant from Kikkoman Corporation.

## ETHICAL REVIEW

This study was conducted in accordance with the Declaration of Helsinki. This study protocol was reviewed and approved by the Tokyo Medical and Dental University Review Board.

## INFORMED CONSENT

Written informed consent was obtained from all study participants.
